# The Qwantify app dataset: A remote experience sampling study of desire, emotion, and well-being

**DOI:** 10.3389/fpsyg.2022.1054292

**Published:** 2022-11-29

**Authors:** Christine D. Wilson-Mendenhall, Paul Condon, Wendy Hasenkamp, George MacKerron, Karen S. Quigley, Lisa Feldman Barrett

**Affiliations:** ^1^Center for Healthy Minds, University of Wisconsin-Madison, Madison, WI, United States; ^2^Department of Psychology, Southern Oregon University, Ashland, OR, United States; ^3^Mind & Life Institute, Charlottesville, VA, United States; ^4^Department of Economics, University of Sussex, Brighton, United Kingdom; ^5^Department of Psychology, Northeastern University, Boston, MA, United States

**Keywords:** desire, wanting, emotion, well-being, meditation, experience sampling method (ESM)

## Abstract

Experiences of desire—the feeling of wanting to have, do, or experience something—are pervasive and varied. Recent theoretical advances draw attention to characterizing this variation. Thus, this study investigated experiences of desire in everyday life and co-occurring social, physical, and emotional states, including facets of emotional experiences known to be related to well-being (e.g., perceived loneliness and stress). The Qwantify app was designed to run a remote experience sampling study. Through the app, participants were randomly alerted during their daily life to report on their experience in the moment. During the data collection period, any individual could download the freely available Qwantify app and participate in the study, without providing identifying information or communicating with researchers. Similar to other remote experience sampling studies, an incentive for participants to engage in the study was unlocking visualizations of their own data. Over 600 participants downloaded the app, completed the sign-up process, and responded to at least one experience sampling alert. Approximately 40% of these participants went on to respond to 50 alerts. The purpose of this report is to describe this experience sampling dataset such that it can be used to test a variety of hypotheses, including hypotheses regarding individual differences.

## Introduction

Desire is typically defined as the subjective experience of motivation—a feeling of wanting that, in its most intense form, is experienced as craving (Kavanagh et al., 2005; Hofmann and Van Dillen, 2012). Over the course of a day, one’s desires might range from wanting a cup of coffee to wanting to see friends or achieve career success. Recent theoretical advances in affective science emphasize investigating these daily life experiences to understand how they vary within and across individuals (Barrett, 2013, 2017; Wilson-Mendenhall, 2017). Thus, this study investigated the desires that people experience during everyday life and co-occurring social, physical, and emotional states. Here we describe the dataset generated by this study so that others can use it to test hypotheses.

Desire has emerged as a topic of study across several subdisciplines of psychological science. Research on self-control tends to focus on desires that people may feel conflicted about and try to resist in everyday life (e.g., spending, media use; Hofmann et al., 2012). In clinical contexts, intense cravings are the focus in research that aims to improve treatment for addiction (e.g., drug and alchohol addiction; Drummond, 2001; Tiffany and Wray, 2012; Brewer et al., 2014; May et al., 2015; Garland et al., 2019). Research in social psychology, on the other hand, highlights the positive impacts of prosocial desires to improve another’s well-being, which is contrasted with desires focused on benefiting oneself without regard for the well-being of others (Crocker et al., 2017; Stellar et al., 2017). This distinction is also present in Eastern philosophies, especially Buddhist philosophies (e.g., Varela et al., 1991; Gethin, 1998; Dambrun and Ricard, 2011). Whereas materialistic desires are often investigated as the contrast to prosocial desires in psychological science (e.g., Kasser, 2016), Buddhist traditions also draw attention to more abstract desires that reflect rigid, fixed views of oneself as independent from others (e.g., desire to be successful at all costs; Ekman et al., 2005; Dambrun and Ricard, 2011). Building on these frameworks and the different types of desire they involve, this study aimed to investigate the full range of desires that people experience in everyday life.

The current study used the experience sampling method (ESM) to investigate desire during daily life. Affective experience is particularly prone to discrepancies between in-the-moment reports and later retrospective recall (Robinson and Clore, 2002; Shiffman et al., 2008; Conner and Barrett, 2012). Thus, the smartphone app Q*want*ify was developed to assess desire in the moment. Participants who engaged with the Q*want*ify app were randomly alerted during their day to report on what, if anything, they were wanting, the nature of this wanting experience, and co-occurring social, emotional, and physical states.

The Q*want*ify study dataset is unique in several ways. First, this app-based study was designed to be fully remote. Participants did not visit a lab and were not required to communicate with researchers. This approach facilitated disclosure of private desires and emotional experiences.

Second, the experience sampling questions assessed desire using a hybrid approach that included open-ended, categorical, and continuous (Likert scale) approaches. A primary way this design differed from prior research is that instead of providing pre-defined desire categories to endorse (e.g., eat, sleep, media use, leisure), participants were asked to describe what they were wanting in an open-ended manner. This approach ensured that the study captured the potentially wide variety of desires that people experience.

Third, the study concurrently assessed facets of social, physical, and emotional experience. Every time a participant reported on experiences of desire in the moment, they also reported on social context (e.g., interaction with others), physical states (e.g., hungry, tired), and emotional experiences (e.g., general mood, specific emotions). A subset of these questions were designed to assess facets of well-being, including perceived stress and loneliness (Salsman et al., 2013).

Finally, this study is unique in that participants varied in their level of meditation experience, which was assessed alongside standard demographic questions. Initial evidence suggests that mindfulness-based interventions can reduce drug craving (Garland et al., 2019), but much remains to be learned regarding how meditation practice may shape experiences of desire (Ekman et al., 2005). Although causal conclusions cannot be drawn from cross-sectional analysis, exploring relationships between meditation experience and daily experiences of desire in this dataset may seed future research.

## Methods

### Overview

The app-based study ran from October 2016 to July 2018. During that time, any individual could download the Q*want*ify app from Apple’s App Store or Google Play. Upon downloading and opening the app, participants were guided through a series of onboarding steps, including informed consent. Participants were sent 2-5 alerts each day to report on their experience in the moment and were encouraged to respond to 50 alerts. During the study, graphs visualizing aspects of their data unlocked as they responded to more alerts. The Q*want*ify app is no longer active, but extensive documentation can be found in the ‘‘Qwantify Documentation’’ PDF on OSF.^1^

### Recruitment

In October 2016, data collection began with a soft launch through the researchers’ social media networks. In collaboration with the Mind & Life Institute (MLI), the organization that funded the research, the study was then more formally publicized through a press release and social media posts in late 2016 and early 2017. Detailed description of specific recruitment activities is provided in the Supplementary Materials.

Data were collected during sign-up in the app to understand how participants learned about the study. Consistent with known recruitment efforts, participants reported hearing about the study via Twitter/Facebook (30% of participants) and the MLI (28% of participants). Informal sharing also occurred, with 18% of participants indicating that they heard about the study from a Friend/Family. Participants also learned about the study through an internet web search (11%), via another internet source (10%), at a scientific conference (4%), and/or through another source (12%).

### Consent and onboarding

Participants completed four onboarding steps before starting the daily experience sampling (see Supplementary Figure 1). The first step was consenting to participate in the study. In clear language on the initial screen, it was indicated that by tapping “I agree,” the participant understood the nature and purpose of the research, that they were consenting to take part in the study, and that they could withdraw at any time. By tapping “agree,” participants also indicated that they were at least 18 years old and were using their own device. Participants were able to e-mail the detailed consent text to themselves.

Next, participants answered standard demographic questions, and provided information about their life satisfaction, mental health status, and experience with meditation. We acknowledge that some of these questions (e.g., regarding sex, gender, race, and ethnicity), while standard for research at the time, were not as inclusive as they should be and thus may have impacted participation.

Participants then confirmed their alert settings (see Supplementary Figure 1). The default setting was to receive three alerts per day. Participants could adjust the number of alerts they received per day (to 2, 4, or 5 alerts) and the time window in which they received alerts during weekdays and weekends. They could also adjust their alert sound, choosing from a list of 12 sounds. The final step guided participants through the questions that would appear each time they responded to an alert, which are described further in the next section. Instructions accompanied each question to ensure participants understood it prior to starting the study. Once the study started, participants could access the instructions for a question by clicking the i button. Participants could also contact the researchers at any time by selecting “Find out more” on the home screen and then “Email us.”

### Experience sampling design

We use the term “survey” to refer to the set of experience sampling questions that were presented, which are listed in Table 1. Each time participants responded to an alert, they were asked to report on what they had been experiencing just prior to starting the survey and to keep this experience in mind as they moved through the questions. Participants were also cautioned not to respond if they were driving or could not otherwise respond safely.

**TABLE 1:** Experience sampling questions.

Question		Type	Response options or anchors	Contingent
1	Did you want anything?	Categorical	Yes, unsure, no	No
2	What did you want most?	Open	Open text entry	Yes
3	How intense was your experience of wanting it?	Slider	Very weak…moderate…very strong	Yes
4	Did you want to feel … ? (Check all that apply)	Categorical	Alert/awake, calm mind/peaceful, good about yourself, successful/accomplished, acknowledged/liked (by another), in control of your situation, connected (with others), excited/inspired, comfort/relief, good/pleasant in your body, something else[fill in], nothing in particular	Yes
5	What were you doing? (Select your primary activity)	Categorical	Work/school, entertainment/leisure, personal care/chores/errands, travel/commuting, talking/conversation, eating, exercising, sleeping/in bed, something else[fill in]	No
6	Were you with other people?	Categorical	Yes-and interacting with them, yes-but not interacting, no	No
7	How were you feeling?	Slider	Very bad…neutral…very good	No
8	How would you describe your energy level?	Slider	Low…high	No
9	How were you feeling physically? (Check all that apply)	Categorical	Hungry, energized, good/pleasant, discomfort (pain, sick, etc.), tired, something else[fill in], nothing in particular	No
10	Were you feeling any of the following emotions? (Check all that apply)	Categorical	Angry, content, awe/amazed, happy, grateful, sad, anxious, resentful, guilty, enthusiastic, restless, compassionate, something else[fill in], not feeling an emotion	No
11	Was something on your mind that you were thinking about repeatedly?	Slider	Not at all…somewhat…very much	No
12	Were you feeling stressed?	Slider	Not at all…somewhat…very much	No
13	Were you feeling lonely?	Slider	Not at all…somewhat…very much	No
14	How were you feeling about yourself?	Slider	Very bad…neutral…very good	No
15	Were you appreciating/enjoying what was happening around you?	Slider	Not at all…somewhat…very much	No

## Desire

The first block of questions was designed to assess desire (Table 1 Q1–4). The first question asked if participants had been wanting anything (yes, no, unsure). If participants selected “yes,” they responded to three other questions. First, they responded to the open-ended question “what did you want most?” by typing a response or using the dictation capability on their phone. They then rated the intensity of their wanting experience on a slider scale from very weak to very strong, with moderate as the midpoint. Finally, they were asked about how they wanted to feel; that is, how they would feel if they could have what they described wanting. Participants could select as many of the 10 affective themes that applied (e.g., calm mind/peaceful, good/pleasant in your body, connected with others; see Table 1), enter in “something else” not on the list, or indicate that they did not want to feel anything in particular. This “want to feel” question was also presented if participants indicated being “unsure” if they wanted anything initially (in Q1), in case an affective theme was present. For questions like this one, which have categorical response options, the response options were initially randomized for each individual participant, but then the order remained fixed for that participant to reduce burden.

## Context

The second block of questions was designed to assess the social, affective, and physical context of that moment (Table 1 Q5–9). Participants were first asked to indicate what they were doing by selecting their primary activity (e.g., work/school, eating, entertainment/leisure; see Table 1 for all options) (Killingsworth and Gilbert, 2010). Participants then indicated whether other people were around and if they were interacting with them. Next, they rated how they were feeling on a Likert scale from very bad to very good with neutral as the midpoint (i.e., affective valence) and their energy level (on a Likert scale from low to high). Finally, they indicated how they were feeling physically in their body (e.g., hungry, tired, good/pleasant), checking as many options as applied.

## Emotions

A third block of questions was designed to assess affective experience with greater precision (Table 1 Q10–11). Participants were presented with a list of 12 emotions and asked to select emotion terms that described how they were feeling. They could also enter an emotion word not on the list or indicate that they were not feeling an emotion. In addition to common emotional experiences (e.g., happy, sad), the list included emotions that are typically other-oriented and/or self-transcendent (e.g., compassionate, awe/amazed), emotions that tend to be more self-focused (e.g., resentful, guilty), and emotions that may be related to the subjective experience of desire or lack thereof (e.g., content, restless) (for the complete list, see Table 1). In this block, participants also indicated whether something was on their mind that they were thinking about repeatedly. This question was designed based on a prior experience sampling study showing that repetition is a key feature of worry and rumination (Kircanski et al., 2015). Valence was not embedded in this question (i.e., repetitive *negative* thinking) because valence was assessed in Block 2, and because we did not want to exclude repetitive thinking that may be related to desire and craving and thus not necessarily experienced as negative.

## Well-being

Finally, the questions in the fourth block were designed to assess psychological and emotional well-being (Table 1 Q12–15). Two of the questions were designed to assess established domains of emotional health: perceived stress and loneliness, respectively (Salsman et al., 2013). A third question assessed how participants felt about themselves (from very bad to very good with neutral as the midpoint). This question was included both because of the relationship observed between low levels of self-esteem and symptoms of depression and anxiety (Orth and Robins, 2013; Sowislo and Orth, 2013), and because unstable self-esteem characterized by larger short-term fluctuations around a mean level (i.e., higher standard deviation) has been linked to defensiveness and high reactivity to self-relevant events (Kernis et al., 1993; Seery et al., 2004; Kernis and Lakey, 2008). Finally, a more exploratory question about outward focus of attention—appreciating/enjoying what was going on around oneself (people, objects, events)—was also included in this set of questions. The questions in this block were randomized; that is, the order of these questions was unpredictable every time a participant completed a survey. This randomization ensured that participants were still paying attention as they finished the survey.

### Interactions and incentives in the app

Participants were sent alerts pseudo-randomly during the time period and at the daily frequency selected during sign-up. For each participant, their daily time window (e.g., 8 am–10 pm) was divided by the number of alerts per day (e.g., 3 alerts per day) to create time segments of equal duration (e.g., 3 segments of 4.67 h) (MacKerron and Mourato, 2013). During each time segment, an alert was sent at a randomly scheduled time and remained active until the next alert was sent. Further technical details about the alert notifications are available in the Supplementary material.

There were several key aspects of the home screen, which we briefly review here and which are described in more detail in the Supplementary material. The top of the home screen visualized metrics about participants’ engagement that updated in real-time: alerts completed, response rate, and typical (median) response time (see Supplementary Figure 2). At any point in time, participants could select “My charts” to swipe through 19 graphs of their own data. Initially, the graphs were locked, with only the title and brief description visible. Graphs unlocked as the participant progressed through the study (as in Supplementary Figure 2). Participants could also go to “My data” at any point and download a csv file of their data.

From the home screen, participants could also select “Right now, I want…”, which launched a new survey. This feature allowed participants to record their experience even if they had already responded to an alert during a time segment (i.e., user-initiated instead of alert-initiated).

Participants were also able to access the alert settings that they set during sign-up from the home screen, which they could change at any point. Here participants could also pause or stop the study. If a participant selected stop, a brief exit survey was delivered.

If a participant responded to 50 alerts, they were thanked for participating in the study and several additional graphs unlocked. Participants were also asked to answer a few questions about their experiences in the study. Upon finishing these questions, participants could choose to continue on, re-engaging in the study for as long as they wished, or to end their participation in the study.

## Participants

Of the 817 participants who completed the sign-up, 620 participants (76%) responded to at least one alert during their daily life. Of the 620 participants who engaged in the experience sampling, 241 completed 50 alert-initiated surveys (39%). To further examine engagement, we calculated the total number of alert-initiated ESM surveys that each participant completed. The bimodal distribution of these totals showed an initial peak of participants who completed a few alert-initiated ESM surveys (and then stopped) and a second peak at 50 alerts (see Supplementary Figure 3).

Table 2 shows the descriptive statistics for select demographic variables, which are presented for participants who completed the sign-up (*n* = 817), participants who completed at least one alert-initiated ESM survey (*n* = 620), and participants who responded to 50+ alerts (*n* = 241). The demographic breakdown for the “Sign-up” and “ESM start” groups were similar. Of note is that approximately two thirds of participants indicated their sex as female, over 80% of participants had attained a college degree, and over half of participants reported living in a country outside the US. Median age was in the mid-30s, approximately a quarter of participants identified as Hispanic or Latino, and a similar proportion identified as a racial minority (according to US census race categories). Table 2 shows that the “ESM 50” group tended to be older, less ethnically and racially diverse, and more highly educated. A larger percentage of participants in this group also reported living in the US.

**TABLE 2:** Descriptive statistics for demographic variables.

	Sign-up (*n* = 817)	ESM start (*n* = 620)	ESM 50 (*n* = 241)
**Age**			
Mean (SD)	37 (13)	38 (13)	42 (13)
Median	35	36	41
Range	18–94	18–87	18–87
**Sex, *n* (%)**			
Female	549 (67%)	416 (67%)	170 (71%)
Male	267 (33%)	203 (33%)	71 (29%)
Not reported	1 (<1%)	1 (<1%)	na
**Ethnicity, *n* (%)**			
Hispanic or Latino	191 (23%)	154 (25%)	52 (22%)
Not Hispanic or Latino	626 (77%)	466 (75%)	189 (78%)
**Race, *n* (%)**			
American Indian	17 (2%)	14 (2%)	7 (3%)
Asian	84 (10%)	53 (9%)	14 (6%)
Black or African Americn	27 (3%)	16 (3%)	2 (1%)
Native Hawaiian/Pacific Islander	4 (<1%)	4 (<1%)	1 (<1%)
White	610 (75%)	476 (77%)	200 (83%)
Other	106 (13%)	82 (13%)	27 (11%)
**Level of Education, n (%)**			
Less than high school graduation	17 (2%)	10 (2%)	1 (<1%)
High school graduation	132 (16%)	93 (15%)	37 (15%)
College degree	289 (35%)	214 (34%)	72 (30%)
Postgraduate degree	379 (47%)	303 (49%)	131 (54%)
**Country, *n* (%)**			
U.S.	336 (41%)	264 (43%)	117 (49%)
Other	481 (59%)	356 (57%)	124 (51%)
**Native language, *n* (%)**			
English	420 (51%)	326 (53%)	140 (58%)
Other	397 (49%)	294 (47%)	101 (42%)
Median age began learning English	10	10	11

One implausible age (305) was excluded from descriptive analyses of age. Demographic variables collected but not shown here include occupational status, annual household income, subjective social status, current marital status, and living situation, including whether living with their children and age brackets of children (see documentation on OSF).

Descriptive statistics for the two mental health questions included in the sign-up were similar across the Sign-up, ESM Start, and ESM 50 groups (see Supplementary Table 3). A little over a quarter of participants indicated a history of psychiatric illness and 14% of participants indicated a current addiction.

Finally, over half of the participants reported that they meditate or have a regular contemplative practice in the Sign-up, ESM Start, and ESM 50 groups (see Supplementary Table 4). Notably, a larger proportion of participants in the ESM 50 group reported having meditation experience (73%), as compared to the ESM Start (64%) and Sign-up (57%) groups. For those who indicated having meditated in the Sign-up, ESM Start, and ESM 50 groups, the distributions of frequency of practice, meditation retreat experience, and years of practice were fairly similar. Over 80% of participants indicated that they had been engaging in their practice for a year or longer and over 50% had attended a meditation retreat. Approximately half of these participants indicated infrequent practice of less than once a week (45–54% depending on group), with only ∼10% reporting that they practiced nearly every day.

## Data files on OSF

Six data files generated from this study and a variables codebook reside on OSF: https://osf.io/sxfrx/. In addition to the files that contain the sign-up data and experience sampling data, separate files contain data on participants’ alert settings, the alert notifications that were sent, and the viewing of “My Charts” and “My Data.” Participants were logged in each data file with a coded alphanumeric ID. An orientation to these files, and how they relate to each other, is provided in the Supplementary material. Text data generated in response to open-ended questions is available to researchers by request (see OSF for request process).

## Data availability statement

The datasets presented in this study can be found in online repositories. The names of the repository/repositories and accession number(s) can be found in the article/Supplementary material.

## Ethics statement

The studies involving human participants were reviewed and approved by the Northeastern University Institutional Review Board. The patients/participants provided their written informed consent to participate in this study.

## Author contributions

CW-M, PC, and WH contributed to conception and design of the study. GM, KQ, and LB advised on the design of the study. GM developed the code to implement the Q*want*ify app and structure the data files. CW-M created the documentation for the Q*want*ify app, prepared the data for analysis, and conducted demographic analyses. CW-M wrote the first draft of the manuscript, with input on an outline and analysis plans from PC and WH. All authors contributed to manuscript revision and approved the submitted version.

## Footnotes

1
https://osf.io/sxfrx/

## References

[B1] Barrett L. F. (2013). Psychological construction: The darwinian approach to the science of emotion. *Emot. Rev.* 5 379–389. 10.1177/1754073913489753

[B2] Barrett L. F. (2017). The theory of constructed emotion: An active inference account of interoception and categorization. *Soc. Cogn. Affect. Neurosci.* 12 1–23. 10.1093/scan/nsw154 27798257 PMC5390700

[B3] Brewer J. A. Elwafi H. M. Davis J. H. (2014). Craving to quit: Psychological models and neurobiological mechanisms of mindfulness training as treatment for addictions. *Transl. Issues Psychol. Sci.* 1 70–90. 10.1037/2332-2136.1.S.70 PMC3434285 22642859

[B4] Conner T. S. Barrett L. F. (2012). Trends in ambulatory self-report: The role of momentary experience in psychosomatic medicine. *Psychosom. Med.* 74 327–337. 10.1097/PSY.0b013e3182546f18 22582330 PMC3372543

[B5] Crocker J. Canevello A. Brown A. A. (2017). Social motivation: Costs and benefits of selfishness and otherishness. *Annu. Rev. Psychol.* 68 299–325. 10.1146/annurev-psych-010416-044145 27362501

[B6] Dambrun M. Ricard M. (2011). Self-centeredness and selflessness: A theory of self-based psychological functioning and its consequences for happiness. *Rev. Gen. Psychol.* 15 138–157. 10.1037/a0023059

[B7] Drummond D. C. (2001). Theories of drug craving, ancient and modern. *Addiction* 96 33–46. 10.1046/j.1360-0443.2001.961333.x 11177518

[B8] Ekman P. Davidson R. J. Ricard M. Wallace B. A. (2005). Buddhist and psychological perspectives on emotions and well-being. *Curr. Dir. Psychol. Sci.* 14 59–63.

[B9] Garland E. L. Hanley A. W. Kline A. Cooperman N. A. (2019). Mindfulness-oriented recovery enhancement reduces opioid craving among individuals with opioid use disorder and chronic pain in medication assisted treatment: Ecological momentary assessments from a stage 1 randomized controlled trial. *Drug Alcohol Depend.* 203 61–65. 10.1016/j.drugalcdep.2019.07.007 31404850 PMC6939880

[B10] Gethin R. (1998). *The foundations of buddhism.* New York, NY: Oxford University Press.

[B11] Hofmann W. Van Dillen L. (2012). Desire: The new hot spot in self-control research. *Curr. Dir. Psychol. Sci.* 21 317–322. 10.1177/0963721412453587

[B12] Hofmann W. Vohs K. D. Baumeister R. F. (2012). What people desire, feel conflicted about, and try to resist in everyday life. *Psychol. Sci.* 23 582–588. 10.1177/0956797612437426 22547657

[B13] Kasser T. (2016). Materialistic values and goals. *Annu. Rev. Psychol.* 67 489–514. 10.1146/annurev-psych-122414-033344 26273896

[B14] Kavanagh D. J. Andrade J. May J. (2005). Imaginary relish and exquisite torture: The elaborated intrusion theory of desire. *Psychol. Rev.* 112 446–467. 10.1037/0033-295X.112.2.446 15783293

[B15] Kernis M. H. Cornell D. P. Sun C. R. Berry A. Harlow T. (1993). There’s more to self-esteem than whether it is high or low: The importance of stability of self-esteem. *J. Pers. Soc. Psychol.* 65 1190–1204. 10.1037//0022-3514.65.6.1190 8295118

[B16] Kernis M. H. Lakey C. E. (2008). Secure versus fragile high self – esteem as a predictor of verbal defensiveness: Converging findings across three different markers. *J. Pers.* 76 477–512. 10.1111/j.1467-6494.2008.00493.x 18447858

[B17] Killingsworth M. A. Gilbert D. T. (2010). A wandering mind is an unhappy mind. *Science* 330:932. 10.1126/science.1192439 21071660

[B18] Kircanski K. Thompson R. J. Sorenson J. E. Sherdell L. Gotlib I. H. (2015). Rumination and worry in daily life: Examining the naturalistic validity of theoretical constructs. *Clin. Psychol. Sci.* 3 926–939. 10.1177/2167702614566603 26783506 PMC4714789

[B19] MacKerron G. Mourato S. (2013). Happiness is greater in natural environments. *Glob. Environ. Change* 23 992–1000. 10.1016/j.gloenvcha.2013.03.010

[B20] May J. Kavanagh D. J. Andrade J. (2015). The elaborated intrusion theory of desire: A 10-year retrospective and implications for addiction treatments. *Addict. Behav.* 44 29–34. 10.1016/j.addbeh.2014.09.016 25306214

[B21] Orth U. Robins R. W. (2013). Understanding the link between low self-esteem and depression. *Curr. Dir. Psychol. Sci.* 22 455–460. 10.1177/0963721413492763

[B22] Robinson M. D. Clore G. L. (2002). Belief and feeling: Evidence for an accessibility model of emotional self-report. *Psychol. Bull.* 128 934–960. 10.1037/0033-2909.128.6.934 12405138

[B23] Salsman J. M. Butt Z. Pilkonis P. A. Cyranowski J. M. Zill N. Hendrie H. C. (2013). Emotion assessment using the NIH toolbox. *Neurology* 80 S76–S86. 10.1212/WNL.0b013e3182872e11 23479549 PMC3662334

[B24] Seery M. D. Blascovich J. Weisbuch M. Vick S. B. (2004). The relationship between self-esteem level, self-esteem stability, and cardiovascular reactions to performance feedback. *J. Pers. Soc. Psychol.* 87 133–145. 10.1037/0022-3514.87.1.133 15250798

[B25] Shiffman S. Stone A. A. Hufford M. R. (2008). Ecological momentary assessment. *Annu. Rev. Clin. Psychol.* 4 1–32. 10.1146/annurev.clinpsy.3.022806.091415 18509902

[B26] Sowislo J. F. Orth U. (2013). Does low self-esteem predict depression and anxiety? A meta-analysis of longitudinal studies. *Psychol. Bull.* 139 213–240. 10.1037/a0028931 22730921

[B27] Stellar J. E. Gordon A. M. Piff P. K. Cordaro D. Anderson C. L. Bai Y. (2017). Self-transcendent emotions and their social functions: Compassion, gratitude, and awe bind us to others through prosociality. *Emot. Rev.* 9 200–207. 10.1177/1754073916684557

[B28] Tiffany S. T. Wray J. M. (2012). The clinical significance of drug craving. *Ann. N. Y. Acad. Sci.* 1248 1–17. 10.1111/j.1749-6632.2011.06298.x 22172057 PMC4041083

[B29] Varela F. J. Thompson E. Rosch E. (1991). *The embodied mind.* Cambridge, MA: MIT Press.

[B30] Wilson-Mendenhall C. D. (2017). Constructing emotion through simulation. *Curr. Opin. Psychol.* 17 189–194. 10.1016/j.copsyc.2017.07.015 28830034

