# Supplementary material for: The Qwantify app dataset: A remote experience sampling study of desire, emotion, and well-being
Source: Front Psychol. 2022 Nov 29;13:1054292. doi: 10.3389/fpsyg.2022.1054292 (PMC9745310; doi:10.3389/fpsyg.2022.1054292)
Supplement: Supplementary file 1 [file Data_Sheet_1.pdf]

Supplementary Materials

Methods

The Q*want*ify app and accompanying website are documented extensively in the Qwantify Documentation on OSF: https://osf.io/sxfrx/. All app screens that correspond to the methodological details and features described here can be viewed in this documentation.

Recruitment

In preparation for data collection, two versions of a short, engaging video on the study were created that could easily be shared on social media (see OSF for the videos). A website was also created that provided an introduction to the study and links to the app. After several rounds of iterative piloting (e.g., to detect bugs, finalize study procedures), data collection began with a soft launch in October 2016. For this soft launch, the researchers shared the recruitment video with their personal networks on social media.

In collaboration with the Mind & Life Institute (MLI), the organization that funded the research, the study was more formally publicized in late 2016 and early 2017. Business cards with information about the study were included in MLI conference bags, a press release was issued about the study, and the video was shared on MLI social media accounts. Throughout 2017 and into 2018, MLI periodically posted about the study on their social media. Other known recruitment activities include a Northeastern University blog post on the study, and a social media post by the Center for Healthy Minds at the University of Wisconsin-Madison.

The convenience sampling strategy used here likely contributed to a final sample with disproportionate representation of certain demographic categories as compared to U.S. census estimates (e.g., larger proportion with postgraduate degrees, smaller proportion who identified as Black or African American, smaller proportion who identified as male). Findings from this dataset should be replicated in future research with samples that are representative of the populations being studied, and care should be taken when generalizing from this dataset.

Alert Notifications

Each alert appeared as the notification "What do you want right now? Please tell us as soon as you safely can." and was accompanied by the sound selected during sign-up. Selecting the notification initiated a survey in the Q*want*ify app. At the same time that the notification was sent, a badge also appeared on the Q*want*ify app (as viewed on the participant's home screen). The badge remained until a survey was completed. If a badge yoked to an alert was present, a survey would initiate upon opening the app. The alert remained active for the participant to respond to until the next alert was sent.

Supplementary Figure 1

Onboarding steps and the alert settings screen.

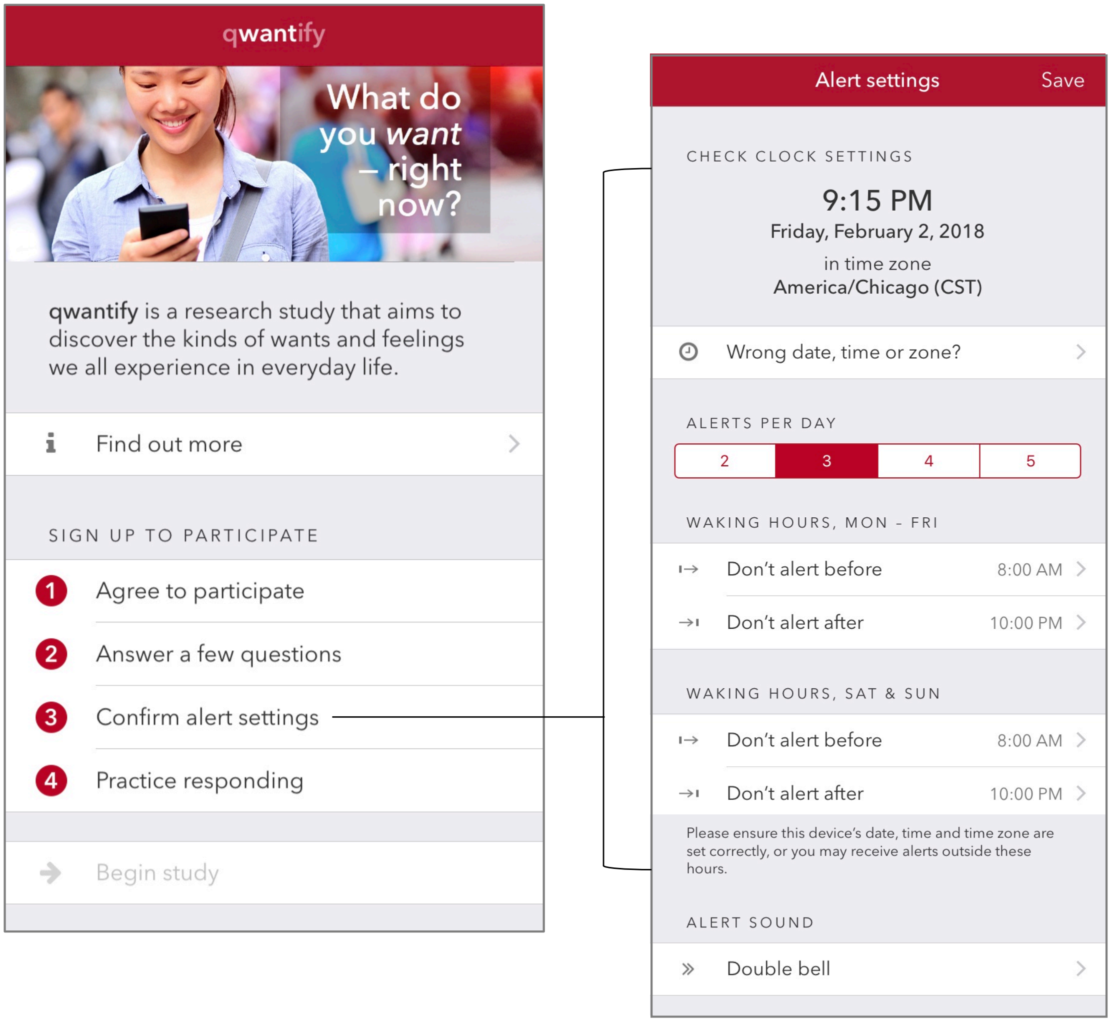

Visualized Engagement Metrics and Personalized Graphs

During the study, the top of the home screen visualized metrics about participants' engagement in the experience sampling that updated in real-time: alerts completed, response rate, and typical response time (see Supplementary Figure 2). The "alerts completed" refers to the cumulative number of alerts that a participant had responded to (by completing the survey questions) at that point in time. Response rate refers to the percentage of alerts that a participant had responded to at that point in time. Typical response time refers to the median time to respond to alerts. These visualizations were designed to motivate participants to respond to alerts as frequently and quickly as possible. Descriptions of these metrics were available to participants through "Find out more" on the home screen (and then clicking "What are the main screen stats?").

The other key interactions through the home screen were "My charts" and "My data" (see Supplementary Figure 2). By navigating to "My charts," participants could swipe through up to 19 graphs of their data. Initially, the graphs were locked, with only the title and description of the graph available for viewing. Graphs unlocked as the participant progressed through the study, with one or more graphs unlocking after responding to 4, 9, 14, 19, 24, 29, 34, 39, 44, and 50 alerts (see Supplementary Figure 2 for examples of unlocked charts). Participants also received notes of encouragement throughout the study (e.g., "You're well on your way with 5 surveys finished…"; "Nice job! That's 10 surveys completed"). Once a graph was unlocked, it continually updated as the participant responded to more alerts and generated more data. At any point in time, participants could also go to "My data" on the home screen and download a csv file of their data.

Exit and End-of-study Procedures

If a participant did not respond to several consecutive alerts, a "we haven't heard from you in a while" notification was sent. This notification asked if the participant wanted to change their alert settings. Upon selecting the notification, the participant was directed to Alert Settings in the app where they could pause or stop the study.

If a participant elected to stop the study at any point, an exit survey appeared asking why they were choosing to stop, with the response options of "I'm too busy", "I'm bored", "I didn't get the data I wanted", or "Something else". If there was anything else the participant wanted the researchers to know, they also had the opportunity to write it in a text box.

Upon responding to 50 alerts, participants were asked to answer a few questions about their experiences in the study. These open-ended questions asked: (1) whether the participant learned anything about themself during the study; (2) whether any of the experience sampling questions seemed odd, difficult, or confusing; (3) if anything highly unusual happened that would cause the participant's data not to represent their normal daily life; and (4) if there was anything else that the participant wanted to share about the study.

Supplementary Figure 2

Home screen of the app, including feedback metrics (left), and examples of graphs in "My charts" that unlocked during the study (right).

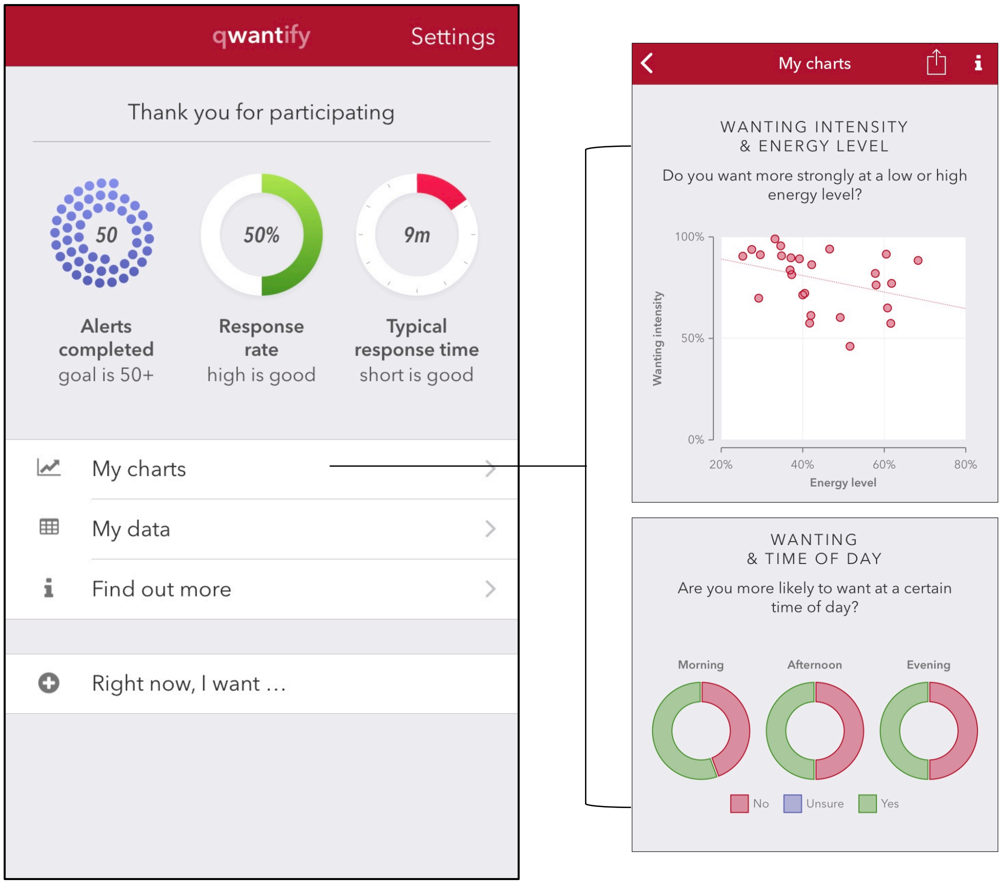

Supplementary Figure 3

Rain cloud plot of the total number of alert-initiated surveys that each participant completed. Individual data points are shown as the violet circles below (with each data point representing a participant). Some participants chose to keep using the app and thus completed more than 50 alert-initiated surveys. For this visualization, any total over 50 alerts was coded as 50 to mark participants who finished the study as it was designed.

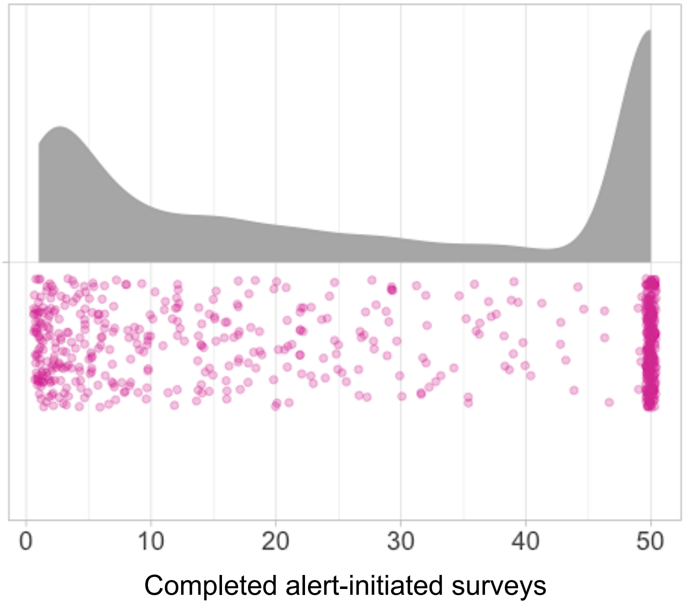

Supplementary Table 3

Descriptive statistics of mental health variables.

	Sign-up *n* = 817	ESM Start *n* = 620	ESM 50 *n* = 241
Current Addiction, n (%)			
Current Addiction	112 (14%)	92 (14%)	34 (14%)
No Current Addiction	676 (83%)	506 (82%)	200 (83%)
Prefer not to say	29 (3%)	22 (4%)	7 (3%)
Mental Health History, n (%)			
History Psychiatric Illness	221 (27%)	185 (30%)	68 (28%)
No History Psychiatric Illness	568 (70%)	417 (67%)	162 (67%)
Prefer not to say	28 (3%)	18 (3%)	11 (5%)

Note. If participants indicated a current addiction and/or history of psychiatric illness, they were asked to briefly describe the nature of the addiction and/or mental health history; this qualitative data has been de-identified and is available to researchers by request.

Supplementary Table 4

Descriptive statistics of meditation experience variables.

	Sign-up<br>*n* = 817	ESM Start<br>*n* = 620	ESM 50<br>*n* = 241
Meditation Experience, n (%)			
Yes	467 (57%)	394 (64%)	176 (73%)
No	350 (43%)	226 (36%)	65 (27%)
Subset Meditation Experience	*n* = 467	*n* = 394	*n* = 176
Frequency, n (%)			
Every day/nearly every day	43 (9%)	35 (9%)	17 (10%)
2 - 4 times per week	73 (16%)	57 (14%)	20 (11%)
About once a week	138 (30%)	114 (29%)	44 (25%)
Less than once a week	213 (45%)	188 (48%)	95 (54%)
Meditation Retreat, n (%)			
Yes	241 (52%)	209 (53%)	95 (54%)
No	226 (48%)	185 (47%)	81 (46%)
Years of Practice, n (%)			
One year or more	387 (83%)	327 (83%)	147 (84%)
Median years of practice	5	5	5
IQR years of practice	8	8	9
Less than one year	76 (16%)	64 (16%)	27 (15%)
Unclear or missing	4 (1%)	3 (1%)	2 (1%)

Note. Several cases in the dataset had a value corresponding to "no" for the initial meditation experience question (i.e., they did not have experience with meditation), but then had values for the questions that should have only appeared in the case of a "yes" to the meditation experience question. Although it is not definitively known why this occurred, it is possible that these participants initially responded "yes" to the meditation experience question and answered some subsequent questions, but then went back and changed their meditation experience response to "no." To be conservative, we removed these cases before calculating the descriptive statistics under "subset meditation experience" above. Other data on meditation practice that was collected, but not displayed here, includes length of practices (i.e., less than 15 min, 15-30 min, 30+ min), approximate number of hours of retreat practice, physical activities during meditation, and mental or emotional practices during meditation (see documentation on OSF).

Data Files and Cleaning

Participants were not asked to provide identifying information such as name or email, and were logged in all data files via a coded alphanumeric ID. The six data files generated from this study and the variables codebook reside on the Open Science Framework (OSF): https://osf.io/sxfrx/.

The *signup* file contains all the demographic and other individual-level data (e.g., meditation experience) logged during Step 2 of the onboarding process. Each row in this file corresponds to an individual participant. As per the research consent, participants who indicated that their age was less than 18 years old in the *signup* file were removed from all data files. One participant contacted the researchers and requested to be removed from the dataset; this individual's data was also removed.

The alert settings information specified during Step 3 of the onboarding is logged in the *alert_settings* file. This file includes the participant's settings at sign-up and also logged any changes the participant made to those settings during the study and/or if the participant paused or stopped the study. This file also includes the exit survey data that was generated if a participant decided to stop the study.

The *alerts_sent* file contains data regarding the alert notifications that participants received, prompting them to complete an experience sampling survey. Each row in this file logs data for an alert notification sent to a participant. Of note is that the ID for the alert notification in this file is recorded in the "record_id" column. If a participant responded to the alert and completed a survey, this ID was logged in the *esm* file (see below) in the "inResponseToAlert_id" column.

The *esm* file is the data generated every time a participant completed a survey, answering the series of experience sampling questions. Each row logs one experience sampling survey completed by a participant. For example, if a participant responded to three alerts, then the participant would have three corresponding rows of survey data in the *esm* file. A row of survey data was also logged each time a participant selected "Right now, I want..." on the home screen and then completed the experience sampling questions. For these rows of data, the "inResponseToAlert_id" column is blank because the survey was not completed in response to an alert notification (i.e., it was not an alert-initiated survey). If a researcher wants to limit their analysis to alert-initiated surveys, it will be necessary to first filter out these rows in which the "inResponseToAlert_id" column is blank.

Finally, the *charts_viewed* file logs a time stamp as a row of data each time a participant viewed "My charts" in the app, where the graphs of their data were located. Similarly, the *data_viewed* file logs a time stamp each time a participant viewed "My data" in the app, where the participant's data could be downloaded.

The raw data files were cleaned prior to posting on OSF, primarily to remove data acquired before the launch of the study (during technical and pilot testing) and to remove specific

individuals' data as described above, due to age or by request. To align with HIPPA de-identification standards, we also replaced U.S. participants' zip code with state in the *signup* file.

Text data generated in response to open-ended questions in the *signup* and *esm* files is available to researchers by request (see OSF for request process). In anticipation of receiving requests for this data, the authors reviewed all of the responses to open-ended questions for information that could potentially be identifying. To prevent any participant from being inadvertently identified, person names in the data that did not clearly refer to a public figure were replaced with the generic code !name!. Place names that could identify a location more specific than state level (e.g., referring to a specific city, university, park, restaurant) were replaced with the generic code !place!. A few other codes embedded in text data are present because they were necessary to accurately read the text data into R: commas were replaced with !c!, apostrophes with !a!, and the number symbol (#) with !n!.
